# Cardioneuroablation for treatment of atrioventricular block: to cure the patient or the electrocardiogram?

**DOI:** 10.1093/europace/euae156

**Published:** 2024-07-02

**Authors:** Jean-Claude Deharo, Artur Fedorowski, Michele Brignole

**Affiliations:** Assistance Publique—Hôpitaux de Marseille, Centre Hospitalier Universitaire La Timone, Service de Cardiologie, France and Aix Marseille Université, C2VN, Cardiologie—Hôpital La Timone, Boulevard Jean Moulin, 13005 Marseille, France; Department of Cardiology, Karolinska University Hospital, Stockholm, Sweden; Department of Medicine, Karolinska Institute, 171 64 Stockholm, Sweden; Department of Clinical Sciences, Lund University, 214 28 Malmö, Sweden; Department of Cardiology, IRCCS Istituto Auxologico Italiano, Faint and Fall Research Centre, S. Luca Hospital, Piazzale Brescia 20, 20149 Milano, Italy

## Abstract

**Graphical Abstract:**
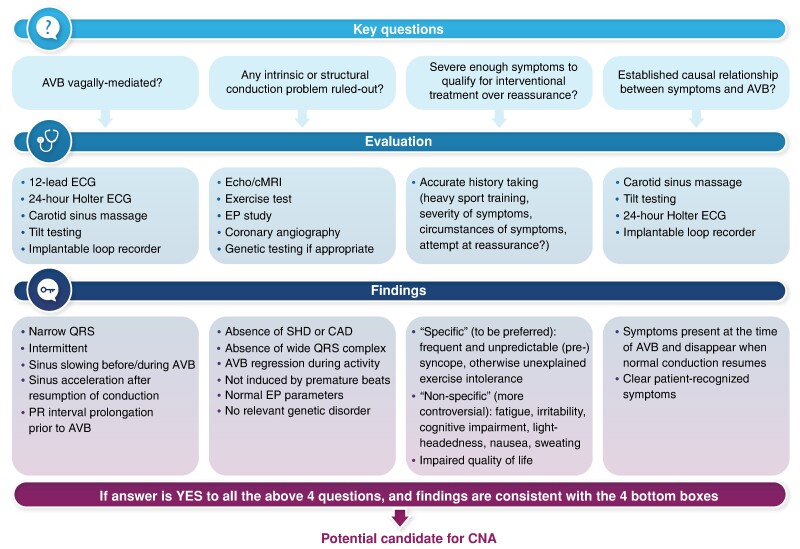

In this issue of the journal, Aksu *et al*.^1^ report the results of a retrospective multicentre registry evaluating cardioneuroablation (CNA) for the treatment of atrioventricular block (AVB). Included patients had second- or higher-degree diurnal AVB, excluding Mobitz II type, deemed to be vagally induced. Atrioventricular block was persistent in 11% of cases, and on top of non-specific symptoms, all the patients except five had experienced syncope. The ablation procedure involved the right or left ganglia was guided by electroanatomical mapping and used extracardiac vagal stimulation in some cases. The primary endpoint was the absence of syncope and elimination of second- or higher-degree diurnal AVB during follow-up. The study population was relatively young: a total of 130 patients were included, with a median age of 34 years. Ablation procedures were effective in 96.2% of cases, with no major procedure-related complications. Of the five patients who failed to achieve the periprocedural result i.e. abortion of AV block, all received or already had a pacemaker. Median follow-up was 300 days. Recurrences were observed, despite achieving immediate success criteria, in 14% of cases. The use of intraprocedural vagal stimulation did not influence the primary endpoint. There was a tendency to achieve an immediate post-procedural success more often when the operator was more experienced. The existence of persistent AVB, older age, and comorbidities including atrial fibrillation, hypertension, and coronary artery disease was associated with the negative primary outcome i.e. syncope or higher-degree AV block recurrence.

The use of CNA for the treatment of AVB had been raised by earlier publications and merited multicentre evaluation.^2,3^ An increasing interest in this topic exists among electrophysiologists as CNA has shown promising results in treating very symptomatic patients with cardioinhibitory reflex syncope.^4,5^ The results of this relatively large registry therefore worth our attention. Nevertheless, as it stands, this report may generate ideas, but its outcomes should be treated with caution and cannot form the basis for expanded CNA recommendations.

Main reasons for being careful about PIRECNA registry results are related to the methodology of the present registry as follows:

1. It is a retrospective, multicentre registry involving 20 centres. The population, the inclusion criteria, the methodology of CNA, the accuracy in the assessment of symptoms, and the documentation of AVB before and during the follow-up are extremely heterogeneous and questionable, making interpretation of results uncertain. In particular, the reader has not been offered any adequate information about possible traumatic incidents, and symptom impact on the quality of life prior to decision to perform CNA. Further, the reason for ablation was non-specific symptoms including fatigue, irritability, lassitude, inability to concentrate, lack of interest, forgetfulness, dizziness feeling of warmth, light-headedness, dizziness, nausea, and sweating. These symptoms are hardly explained by intermittent AV block. In clinical practice, these patients do not appear to be common, and there is a risk of significant over-interpretation of such non-specific symptoms and their possible association with an intermittent conduction disorder often seen in otherwise young and trained people.
2. There is a discrepancy between the inclusion criteria, i.e. 2nd degree or advanced AV block and symptoms of ‘fatigue, irritability, lassitude, inability to concentrate, lack of interest, forgetfulness, dizziness feeling of warmth, light-headedness, dizziness, nausea, sweating, or syncope’, and the primary outcome, i.e. freedom from syncope and of any symptomatic diurnal AVB. The primary endpoint should have been consistent with the criteria used for patient inclusion, i.e. not only to demonstrate the disappearance of AV block but also to assess the improvement of subjective symptoms that led to decision to perform the procedure. In the absence of proven symptom improvement, CNA may cure the ECG, but not the patient.
3. From a pathophysiological point of view, it is very difficult to exclude an underlying intrinsic abnormality of AV node conduction. The normal outcome of electrophysiological study and the improvement of AV conduction after atropine infusion are not sufficient to exclude an intrinsic abnormality. An alternative and likely explanation is that vagal output simply unmasks an underlying AV node disease. This reasoning has important clinical consequences. Cardioneuroablation does not eliminate a conduction system disease, but rather eliminates the ECG manifestations of a concealed intrinsic AV node disease that remain as it is and will likely progress in the future to more severe forms.

Considering the risk of a widespread use of this method in patients with undefined AV block aetiology and non-specific symptoms, we recommend following a clear diagnostic pathway for adequate selection of CNA candidates (*Graphical Abstract*). The aim of this pathway is to make it highly probable that the conductive disorder is vagal in nature, and that it is involved in severe symptoms with a clear cause-and-effect relationship.

1. Demonstration of the direct involvement of the parasympathetic system in the conduction disturbance is key. The characteristics of AVB episodes must be carefully analysed either during spontaneous episodes captured by prolonged ECG monitoring or during provocative tests. It is clear that a high vagal tone may be a cause of bradycardia, particularly in young and trained individuals.^6–8^ In these patients, there is usually permanent sinus bradycardia, on top of which episodes of paroxysmal first- or second-degree AVB of the Mobitz 1 type, or even more advanced second degree and complete AV block may occur. The analysis of sinus rate before the occurrence of the AV block is the clue for differentiating vagal AVB from intrinsic AVB.^6,7,9^ Slowing of the sinus node rate characterizes vagal AVB while the intrinsic form is generally precipitated by an increase in sinus rate and/or premature ventricular contractions. An increase of PR interval before the occurrence of blocked *P* waves is also the rule to follow before episodes can be classified as vagal AV block. The improvement of the AV node conduction properties after administration of atropine is well known and does not really help in decision-taking from a clinical point of view since vagal AVB is usually transient making the evaluation of the effect of atropine on the block unreliable as a clinical indicator.^10^ Intrinsic nodal AVB also respond to atropine, although a lesser extent, and there is no clear cut-off between intrinsic and extrinsic AVB. The most recent guidelines on cardiac pacing clearly advocate for pacing in Mobitz I AV block when symptoms are related to the AVB or the localization of the conduction disturbance is below the AV node. In fact, in more advanced AV block, vagal AV block should be recognized to avoid pacing.^6,7^
2. Any intrinsic or structural conduction problem should be ruled out by using the most appropriate tests. Rarely, vagal syncope or other types of reflex syncope are accompanied by paroxysmal AVB.^11^ If we were to consider an intervention for paroxysmal AVB, the characteristics in favour of vagal AVB are generally considered as indicatory for a non-interventional management.^6,7,9,12^
3. The symptoms must be severe enough to qualify for interventional therapy. Since hospital- and home-monitoring systems are on the rise, we see a danger in treating conduction abnormalities that are not related to a serious disease and are associated with non-specific symptoms. We recommend relying on symptoms that are specific of bradycardia rather than on non-specific symptoms. Quality of life should be clearly impaired.
4. Symptoms should be clearly related to the electrocardiographic abnormality. Due to the episodic nature of conduction disturbances, provocative tests are recommended as the first step like in the evaluation of unexplained syncope.^13,14^ The role of implantable loop recorder is important in inconclusive cases and should probably be part of the evaluation in most patients before decision to embark on interventional pathway.^15^

## Conclusion

Since vagally induced AV block is often a benign condition, its interventional therapy should be aimed at symptom improvement in severely affected patients, in whom a clear cause–effect relationship can be established. Although the long-term effects of CNA are unknown, this method may seem attractive. However, well-designed trials should be performed before it is recommended for wider use. Until the results of such studies are available, we recommend to follow the official position of a recent joint document^16^ issued by the principal arrhythmia societies that state: ‘in patients with extrinsic (functional) sinus node disease and AV block, CNA should be treated as investigational, and if considered in severely symptomatic patients, after proven failure of non-invasive conventional therapies, it requires the setting of controlled trials’.
